# A Novel Thermosensitive Curcumin-Loaded Hydrogel That Modulates Macrophage M1/M2 Polarization for Osteoarthritis Therapy

**DOI:** 10.3390/gels12010007

**Published:** 2025-12-21

**Authors:** Yuanyuan Zhou, Shengsheng Li, Zitong Huang, Zhongjia Yu, Hang Liu, Wanshan Wu, Qiao Xu, Keyun Chen, Jun Huang

**Affiliations:** 1 School of Agriculture and Bioengineering, Foshan University, Foshan 528225, China; 2 School of Animal Science and Technology, Foshan University, Foshan 528225, China; 3 Institute of Biological and Medical Engineering, Guangdong Academy of Sciences & National Engineering Research Center for Healthcare Devices, Guangzhou 510632, China

**Keywords:** osteoarthritis, thermo-sensitivity, hydrogel, curcumin, macrophage

## Abstract

Osteoarthritis (OA) is a degenerative joint disease characterized by cartilage degradation, inflammation, and pain, for which conventional systemic therapies often lack sustained efficacy. Therefore, localized delivery platforms that provide both sustained release and therapeutic activity are urgently needed. We developed a thermosensitive injectable hydrogel—hydroxybutyl chitosan (HBC)—that transitions from a sol to a gel at physiological temperature (37 °C). Curcumin, a natural anti-inflammatory compound with poor bioavailability, was loaded to create a composite hydrogel system (Cur@HBC). HBC exhibited excellent injectability, stability, and biocompatibility. Cur@HBC enabled sustained release of curcumin and significantly attenuated OA progression in vivo, as evidenced by reduced cartilage degradation, decreased expression of MMP13 and pro-inflammatory cytokines (IL-1β, IL-6, TNF-α), improved Collagen II retention, and recovery of cartilage function. Mechanistically, curcumin inhibited chondrocyte apoptosis and promoted macrophage polarization toward the M2 phenotype. This study presents a dual-functional hydrogel platform that combines thermosensitive mechanical support with sustained anti-inflammatory drug delivery. The injectable Cur@HBC hydrogel shows great promise as a localized OA therapy, with the potential to improve joint function.

## 1. Introduction

Osteoarthritis (OA) ranks as the seventh leading cause of disability among individuals over the age of 70 worldwide. The associated pain and disability significantly diminish patients’ quality of life. Epidemiological studies indicate that approximately 50% of individuals aged over 60 exhibit radiographic evidence of OA, with 30–50% experiencing clinical symptoms. Additionally, the Bayesian Age–Period–Cohort (BAPC) model indicates an increasing trend in OA incidence [1]. It is predicted that the years lived with disability (YLD) attributable to OA in China will continue to rise, potentially peaking by 2030 [2]. Moreover, the prolonged course and recurrent episodes consume significant healthcare and nursing resources. Osteoarthritis-related medical expenditures account for approximately 1–2.5% of the gross national product [3]. The global economic impact of early onset osteoarthritis exceeds USD 106.87 billion in direct costs, with nearly 60% attributed to indirect productivity loss. According to the Global Burden of Disease (GBD) study, OA affected 7.6% of the global population (approximately 595 million people) in 2020. This burden has continued to increase globally, affecting around 7.6% of the global population in 2024 [4].

OA is a chronic degenerative joint disease characterized by primary or secondary degeneration and structural disruption of the knee articular cartilage. It involves synovitis, subchondral cartilage exfoliation, osteophyte formation, and impaired subchondral bone remodeling, whose changes gradually compromise joint stability and integrity, ultimately resulting in joint dysfunction, deformity, and chronic pain. Clinically, OA presents as knee pain, swelling, deformity, stiffness, and impaired flexion and extension [5,6,7]. Currently, clinical treatment of OA primarily relies on systemic administration of analgesics such as opioids, steroids, non-steroidal anti-inflammatory drugs (NSAIDs), and acetaminophen to relieve symptoms. Among these, oral and topical NSAIDs (including COX-2 inhibitors) are commonly employed as first-line therapies for osteoarthritis [8]. However, these conventional systemic therapies merely reduce the production of prostaglandin E2 during inflammation or decrease neural sensitivity, thereby providing pain relief without exerting substantial anti-inflammatory or disease-modifying effects [9]. Consequently, they fail to prevent cartilage degradation or address the intra-articular inflammatory microenvironment that underlies OA progression [10]. Furthermore, long-term use of systemic medications is frequently limited by significant side effects such as dependency, gastrointestinal adverse effects, hepatotoxicity, and nephrotoxicity. Therefore, conventional systemic therapies exhibit limited sustained efficacy in chronic OA management, highlighting the urgent need for localized, long-acting therapeutic strategies. Intra-articular corticosteroid injections are commonly used for osteoarthritis management and typically exhibit fewer adverse effects; however, frequent injections still pose risks such as joint infection, nerve damage, and osteoporosis [11].

Numerous studies have proved that inflammation (e.g., synovitis) and cartilage degeneration are critical pathological factors during the early stages of OA. Synovitis persists throughout the entire development of OA, accelerates disease progression, and contributes significantly to joint pain [12]. The synovium secretes cytokines, which recruit numerous macrophages that can differentiate into either M1 or M2 subtypes. For instance, an increased presence of inflammatory M1 macrophages and/or a reduced number of anti-inflammatory M2 macrophages is closely related to cartilage degeneration and the severity of OA. Kou et al. developed artificial opsonin nanoparticles loaded with anti-inflammatory drugs (IgG/Bb@BRPL), which are preferentially internalized by activated M1 macrophages. Through reactive oxygen species (ROS) clearance and inhibition of the NF-κB pathway, these nanoparticles facilitate the re-polarization of macrophages from M1 towards the M2 subtype. This shift helps to restore the metabolic functions of chondrocytes under osteoarthritic conditions, effectively enhancing the therapeutic efficacy by simultaneously suppressing inflammation and promoting cartilage repair. These findings suggest that strategies aimed at converting pro-inflammatory M1 macrophages into anti-inflammatory M2 macrophages are feasible and promising anti-inflammatory therapeutic approaches [13].

In recent years, traditional Chinese medicine (TCM) has demonstrated significant clinical efficacy in the treatment of OA. TCM formulations and monomeric compounds exhibit notable anti-inflammatory effects. Among these, curcumin—a polyphenolic compound extracted from turmeric plants—has garnered considerable attention [14]. Curcumin exhibits anti-inflammatory, antioxidant, and antimicrobial activities [15,16]. Emerging evidence demonstrates that curcumin inhibits key inflammatory signaling pathways; suppresses the production of cytokines such as IL-1β, IL-6, and TNF-α; and downregulates matrix-degrading enzymes including MMP13, thereby attenuating cartilage matrix loss and chondrocyte apoptosis. Thus, curcumin has been widely utilized in the management of OA [17,18,19,20]. Beyond its intrinsic bioactivity, considerable effort has been devoted to enhancing its therapeutic efficacy for OA. One strategy involves developing curcumin delivery systems, including nanomaterials [21], metal–organic frameworks (MOFs) [22], extracellular vesicles [23], and microgels [24], to improve bioavailability and targeting. Another strategy involves investigating the therapeutic mechanisms of curcumin, such as inhibiting inflammation, combating oxidative stress, preventing cell apoptosis, inhibiting angiogenesis, and promoting cartilage regeneration [25,26,27,28]. These findings underscore curcumin’s potential as a multi-target agent capable of regulating inflammation, oxidative stress, and cartilage homeostasis, thus offering a compelling therapeutic avenue for osteoarthritis management. However, curcumin, as a β-diketone polyphenol, is limited by its poor water solubility, chemical instability, and low bioavailability [29]. The development of optimized delivery platforms is, therefore, essential to provide viable strategies to enhance its therapeutic efficacy and advance its application in OA management.

In recent years, injectable hydrogels have emerged as promising intra-articular therapeutic platforms for osteoarthritis due to their excellent biocompatibility, tunable mechanical properties, and ability to undergo in situ gelation [30]. Various natural and synthetic polymers—including hyaluronic acid, alginate, chitosan, and PEG-based derivatives—have been engineered to construct thermosensitive, pH-responsive, and self-healing hydrogel systems capable of modulating the inflammatory microenvironment within the joint. These hydrogels not only provide lubrication and mechanical support but also enable sustained and localized release of anti-inflammatory or antioxidant agents, thereby attenuating cartilage matrix degradation and improving joint function [31]. Moreover, composite systems integrating metal–organic frameworks, nanoparticles, or extracellular vesicles into hydrogel matrices have demonstrated enhanced intra-articular retention and regenerative potential in preclinical models [32,33]. Hydrogel microspheres have been proved to encapsulate drugs effectively, enabling controlled release and sustained drug delivery [34]. J. Li developed a flexible hydrogel composed of a sodium alginate (SA) matrix and curcumin-loaded MOFs, which achieved effective drug loading and sustained release [15]. Collectively, these advances highlight injectable hydrogels as a strategically important direction for overcoming the limitations of conventional pharmacological therapies and achieving precise, localized treatment of osteoarthritis.

In this study, we developed an injectable hydrogel named HBC with thermosensitivity. Native chitosan shows limited solubility at physiological pH and lacks the capacity to form injectable gels, restricting its use in intra-articular delivery. By introducing hydroxybutyl groups, HBC gains excellent aqueous solubility and a reversible thermosensitive sol–gel transition, allowing it to flow during injection and rapidly gel in the joint cavity. This modification enables the formation of an in situ depot that conforms to the joint space and enhances local drug retention, substantially improving its suitability for intra-articular applications. The hydrogel possesses a cross-linked network structure that exists as a flowing liquid at 4 °C and undergoes an in situ phase transition to a stable, supportive gel state at 37 °C. This hydrogel offers advantages in terms of convenient storage and injection, while simultaneously providing lubrication, mechanical support, and cushioning effects after gelation, thereby reducing particulate friction between cartilage surfaces within the joint cavity. On this basis, we further established an injectable thermosensitive hydrogel drug delivery system loaded with curcumin. This study comprehensively evaluated the physicochemical properties, thermosensitive behaviors, sustained release profiles, in vitro and in vivo therapeutic efficacy, and mechanism of this curcumin-loaded hydrogel. Our findings provide a robust foundation for the development of innovative localized OA therapies, aiming to improve therapeutic outcomes, minimize systemic toxicity, and ultimately enhance patients’ quality of life.

## 2. Results and Discussion

### 2.1. Results

#### 2.1.1. Synthesis and Characterization

**Figure 1:**
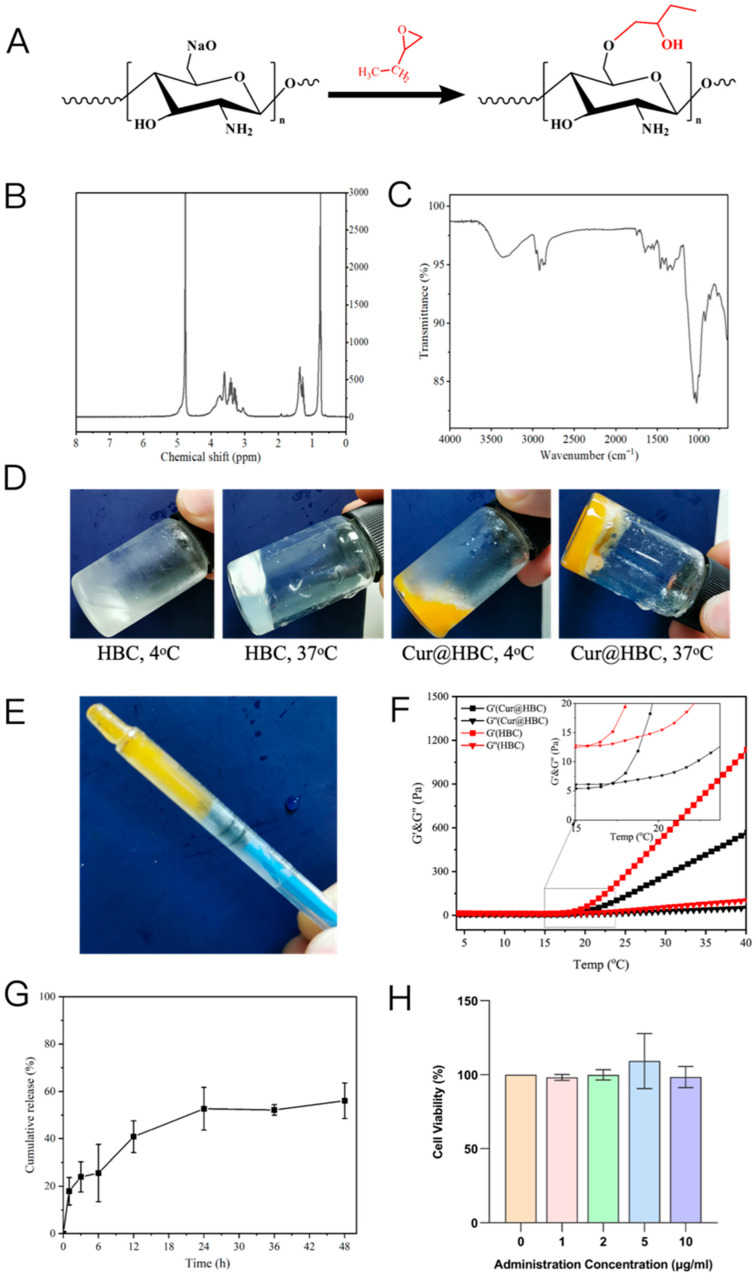
(**A**) Schematic illustration of the modification pathway of hydroxybutyl chitosan (HBC); (**B**) 1H NMR spectrum of HBC; (**C**) FTIR spectra of HBC; (**D**) macroscopic appearance of HBC and Cur@HBC at 4 °C and 37 °C; (**E**) injectable capability of HBC; (**F**) rheological properties of HBC and Cur@HBC determined by oscillatory temperature sweep mode; (**G**) in vitro release profile of curcumin from Cur@HBC in PBS containing 1% Tween-80; (**H**) cell viability of BHK-21 cells after incubation with different concentrations of HBC in a CCK-8 assay.

As shown in Figure 1A, HBC was obtained via alkaline ring-opening grafting of 1,2-epoxy butane onto chitosan. The C6 position is the favored substitution site because its primary hydroxyl is readily deprotonated under basic conditions and is minimally sterically hindered, facilitating ether formation. This positional preference is consistent with established polysaccharide O-alkylation mechanisms [35,36], though partial substitution at secondary positions is expected and intrinsic to polysaccharide chemistry. In the ^1^H NMR, the expected resonances were observed for the N-acetyl group (~1.9 ppm), H-2 (~3.0 ppm), and the non-anomeric ring protons (3.5~4.0 ppm). Aliphatic signals appeared within 0.7~1.7 ppm, and the carbohydrate backbone signals were retained (Figure 1B). The FTIR spectrum exhibited a broad O-H/N-H stretch at 3300–3450 cm^−1^, prominent aliphatic C–H stretches at 2919 and 2873 cm^−1^, and amide bands at ~1647 cm^−1^ and 1576 cm^−1^ (amide). The distinct CH_3_ deformation at ~1462 cm^−1^ and CH_2_/CH_3_ wagging near 1374~1390 cm^−1^ indicate the presence of hydroxybutyl substituents, while the polysaccharide framework is evidenced by C-O-C (glycosidic) stretching at 1146 cm^−1^ together with C-O stretches at 1100~1056 cm^−1^, and a band around 1026 cm^−1^ is assigned to primary-alcohol C-O vibrations on the substituted units (Figure 1C). The degree of substitution (DS) of hydroxybutyl groups was first estimated from the ^1^H NMR spectrum by integrating the terminal methyl resonance at 0.8~1.1 ppm (3H) relative to the normalized glucosamine ring protons (H2–H6, 5H) at 3.0~4.0 ppm, yielding a calculated DS of approximately 1.02. To provide an independent verification, elemental analysis was used to estimate grafting based on the decrease in nitrogen content and the corresponding increase in carbon content after modification. Assuming C_4_H_9_O_2_ as the grafted moiety, the EA-derived DS was approximately 1.25 per repeating unit (Table S1). Although the DS values obtained by NMR and EA were not identical, the difference was small and well within the expected variation for polysaccharide derivatives, where signal overlap, chain heterogeneity, and micro-environmental effects commonly introduce minor discrepancies. The consistency between these two independent measurements confirms that HBC carries roughly one hydroxybutyl substituent per glucosamine unit, which aligns with the known reactivity of chitosan under alkaline O-alkylation conditions. At the macroscopic level, aqueous HBC is a free-flowing sol at 4 °C and forms a self-supporting gel at 37 °C; curcumin-loaded HBC (Cur@HBC) showed the same pattern, defining a practical handling window with cooling before administration and rapid in situ setting under physiological conditions (Figure 1D). Figure 1E reveals good injectability. A short clip documenting the sol–gel transition of Cur@HBC from 4 °C to 37 °C is provided as Supplementary Video S1.

As shown in Figure 1F, the rheological properties of the Cur@HBC system were characterized using an oscillatory temperature sweep mode (4 to 40 °C, 1 Hz, 1% strain, parallel-plate geometry, 20 mm diameter, 1 mm gap). For HBC, the storage modulus (G′) surpassed the loss modulus (G″) at 15.7 °C, indicating the formation of an elastic network. Upon incorporating curcumin, the G′/G″ crossover shifted modestly to 17.25 °C. A reasonable explanation is that curcumin occupies a fraction of association-prone sites within the HBC microenvironment and interacts with hydrophobic/hydrogen-bonding domains, so a slightly higher temperature is required to complete dehydration and achieve network percolation. The increase is small and leaves ample margin for practical application; both HBC and Cur@HBC exist firmly within the gel regime at 37 °C (G′ > G″), which supports depot integrity after placement. The thermoresponsive sol–gel transition of HBC arises from the temperature-dependent balance between hydrogen-bonded hydration and hydrophobic association. At low temperature, extensive hydration stabilizes the polymer chains; upon heating, dehydration and aggregation of hydroxybutyl domains yield a physically crosslinked elastic network. This mechanism corresponds closely to the LCST-type transitions described in recent analyses of hydrogen-bond-regulated thermosensitive polymers [37]. Consistent with these rheological data, the photographs in Figure 1D illustrate that cooling lowers viscosity for smooth passage through fine-gauge needles, whereas body temperature gelation fixes the material at the target site, thereby limiting leakage in confined spaces such as the joint cavity. To assess the distribution of curcumin within the matrix, SEM imaging was performed on freeze-dried HBC and Cur@HBC samples (Figure S1). Both materials exhibited the irregular porous morphology typical of physically crosslinked HBC networks, while Cur@HBC showed uniformly dispersed fine particulates along pore walls, indicating stable micro-scale dispersion rather than aggregation. Release testing in PBS containing 1% Tween-80 showed a burst-then-sustained profile for Cur@HBC: an initially faster phase over the first ~3 h, followed by a marked slowdown approaching quasi-equilibrium by 24 h, with a 48 h cumulative release of 56.06 ± 7.5%. The early phase is attributable to desorption/diffusion of the drug near the gel–medium interface or at weakly bound sites; the slower phase reflects diffusion from the interior network as the matrix equilibrates (Figure 1G). For intra-articular administration, the combination of a low-temperature sol for accurate needle placement, rapid gelation at 37 °C for spatial retention, and day-scale sustained release aligns with the pharmacologic goal of tempering synovial inflammation while minimizing systemic exposure. The modest upward shift in gelation temperature upon drug loading does not hinder performance under physiological conditions, and the physically crosslinked, compliant network accommodates joint motion without brittle failure, helping to stabilize residence and release. To evaluate the biosafety of HBC, we performed a cytotoxicity assay on BHK-21 cells. As shown in Figure 1H, after 24 h culture in the presence of HBC at varying concentrations, the cell survival rate was still above 90% even at 10 mg/mL, indicating its satisfactory cytocompatibility.

#### 2.1.2. Cur@HBC Exerts the Best Performance in Attenuating OA Development in Mice

**Figure 2:**
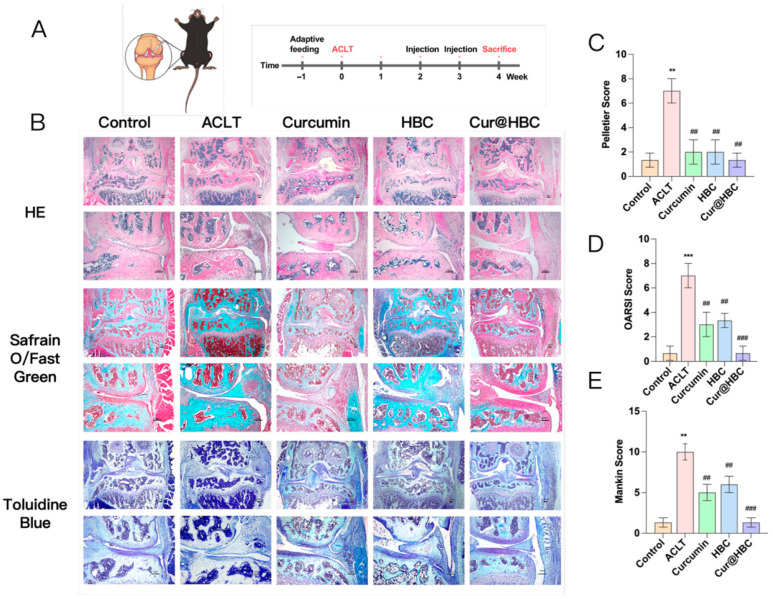
Cur@HBC exerts the best performance in attenuating OA development in mice. (**A**) The treatment protocol for ACLT-induced OA mice with intra-articular injection of saline, curcumin, HBC, or Cur@HBC. (**B**) Representative histological images of knee joints stained with H&E, Safranin O/Fast Green, and Toluidine Blue at week 4. Scale bar = 200 μm. Quantitative scoring of (**C**) Pelletier synovitis score; (**D**) OARSI score for cartilage degeneration; and (**E**) Mankin score for cartilage damage. Data are presented as the mean ± SD (n = 5). ** *p* < 0.01, *** *p* < 0.001 compared with control group; ## *p* < 0.01, ### *p* < 0.001 compared with ACLT group.

An OA mouse model was established via ACLT surgery to test the efficacy of our newly developed drug-loading hydrogel. Histological analyses, including H&E, Safranin O/Green, and Toluidine Blue staining, were performed on the knee joint sections of mice at week 4 post-surgery. As shown in Figure 2B, the ACLT group exhibited marked cartilage erosion, synovial inflammation, and proteoglycan loss compared to the control group. In contrast, treatment with curcumin, HBC, and Cur@HBC substantially preserved cartilage integrity, reduced synovial hyperplasia, and maintained proteoglycan content. Quantitative histopathological scoring further confirmed these findings. Compared to the ACLT group, Cur@HBC treatment significantly reduced the Pelletier inflammation score (Figure 2C), OARSI score (Figure 2D), and Mankin score (Figure 2E). These results suggest that the Cur@HBC composite exhibits superior chondroprotective and anti-inflammatory properties compared to individual treatments.

**Figure 3:**
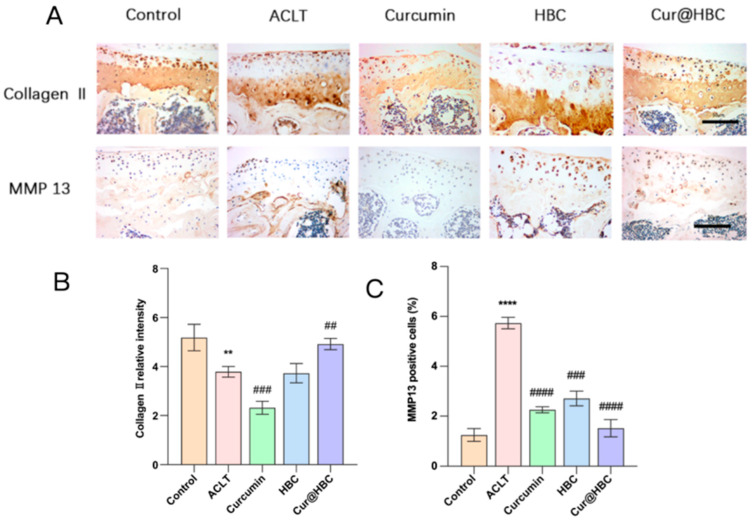
Cur@HBC treatment preserves cartilage matrix and reduces catabolic enzyme expression in osteoarthritis. (**A**) Representative immunohistochemical staining of Collagen II and MMP13 in articular cartilage sections from each group; scale bar = 100 μm. (**B**) Quantitative analysis of Collagen II expression. (**C**) Percentage of MMP13-positive cells. Data are presented as mean ± SD (n = 5). ** *p* < 0.01, and **** *p* < 0.0001 compared with the control group; ## *p* < 0.01, ### *p* < 0.001, and #### *p* < 0.0001 compared with the ACLT group.

Histological analysis revealed that ACLT-induced osteoarthritis led to a marked reduction in Collagen II expression and an increase in MMP13-positive cells in articular cartilage relative to the control group. Treatment with curcumin alone moderately preserved Collagen II and reduced MMP13 expression. Notably, both HBC and Cur@HBC treatments significantly ameliorated cartilage degradation. The Cur@HBC group exhibited the strongest Collagen II staining and the lowest MMP13 expression among all treated groups (Figure 3A). Quantification confirmed these observations. Collagen II relative intensity significantly decreased in the ACLT group, while Cur@HBC treatment significantly restored it (Figure 3B). MMP13-positive cell percentage was significantly elevated in the ACLT group, and this increase was significantly suppressed in the HBC, curcumin, and Cur@HBC groups (Figure 3C).

#### 2.1.3. Cur@HBC Treatment Preserves Joint Structure and Improves Motor Function in ACLT-Induced Osteoarthritis

**Figure 4:**
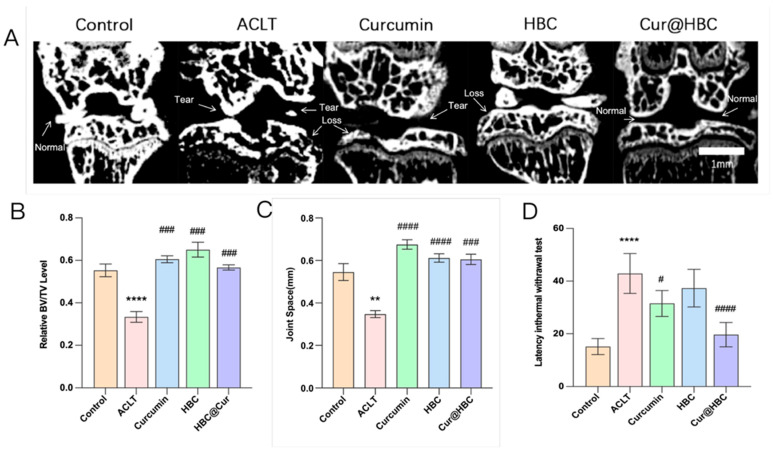
Cur@HBC treatment preserves joint structure and improves motor function in ACLT-induced osteoarthritis. (**A**) Representative micro-CT images of knee joints. Notable features such as cartilage loss, joint space narrowing, and bone tear are marked. Scale bar = 1 mm. (**B**) Quantitative analysis of relative bone volume/tissue volume (BV/TV). (**C**) Joint space measurements (mm). (**D**) Behavioral evaluation of motor function based on “latency in thermal withdrawal test” response (in seconds). Data are presented as mean ± SD. ** *p* < 0.01, and **** *p* < 0.0001 compared with control group; # *p* < 0.05, ### *p* < 0.001, and #### *p* < 0.0001 compared with ACLT group.

Micro-CT imaging revealed pronounced structural damage in the ACLT group, including cartilage loss and joint space narrowing. In contrast, the joints of the Cur@HBC group retained a normal architecture similar to that of the control group (Figure 4A). Quantification of BV/TV showed a significant decrease in the ACLT group, indicating bone loss. While both HBC and curcumin monotherapies showed partial protection, the Cur@HBC combination preserved BV/TV levels close to normal (Figure 4B). Joint space was significantly reduced in the ACLT group, indicating cartilage degeneration. The HBC, curcumin, and Cur@HBC treatments significantly restored joint space width, suggesting improved cartilage preservation (Figure 4C). ACLT surgery resulted in significant motor impairment, as indicated by the increased “latency in thermal withdrawal test” values. All treatments improved performance, with Cur@HBC resulting in the most significant recovery (Figure 4D).

#### 2.1.4. Curcumin Alleviates Chondrocytes Apoptosis and Inflammation and Improves Functional Outcome

**Figure 5:**
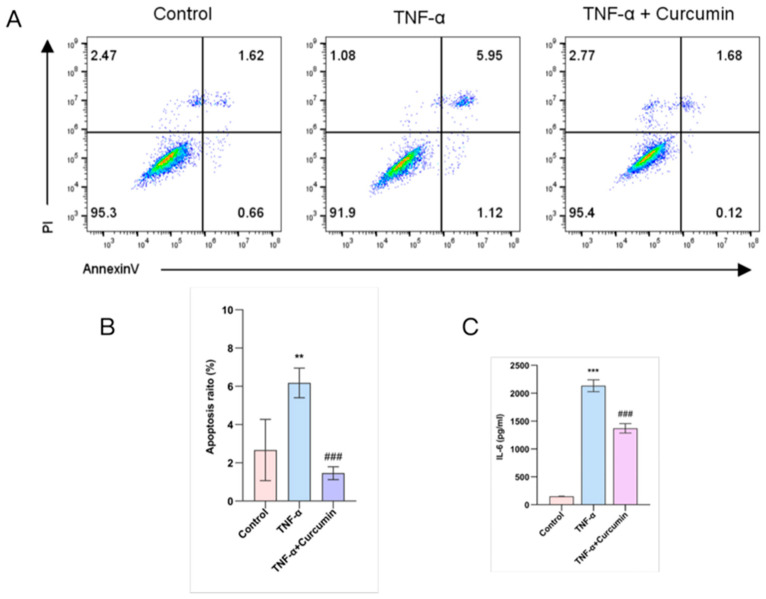
Curcumin alleviates chondrocyte apoptosis and inflammation. (**A**) Flow cytometry analysis of apoptosis in chondrocytes; (**B**) quantification of apoptotic cells from panel A; (**C**) IL-6 levels in chondrocytes measured by ELISA. Data are presented as mean ± SD. ** *p* < 0.01, *** *p* < 0.001, compared with control; ### *p* < 0.001 compared with TNF-α.

Flow cytometric analysis revealed that TNF-α stimulation significantly increased the proportion of apoptotic cells relative to the control. Treatment with curcumin drastically reduced this apoptotic population, indicating its anti-apoptotic effect (Figure 5A,B). TNF-α treatment robustly increased IL-6 production, a key inflammatory cytokine, while curcumin significantly suppressed IL-6 secretion (Figure 5C).

#### 2.1.5. Curcumin Modulates Macrophage Polarization

**Figure 6:**
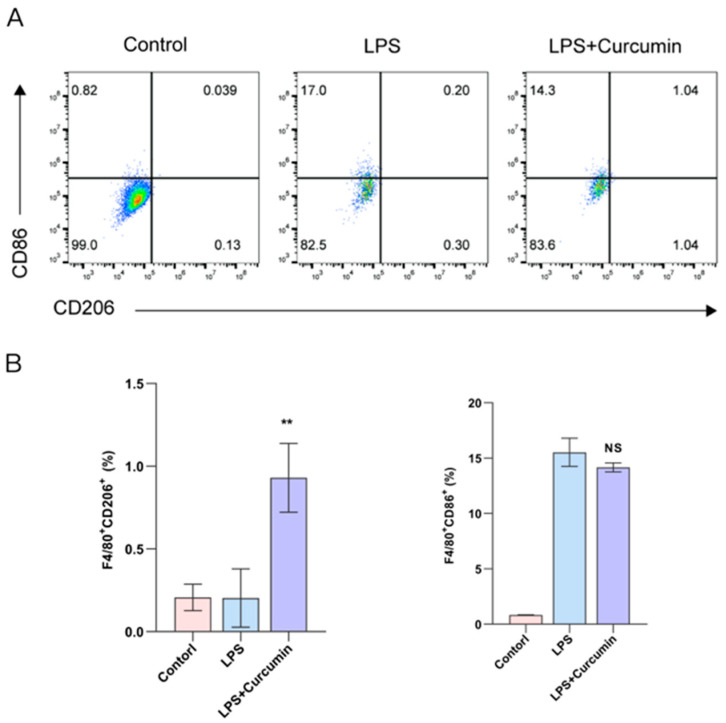
Curcumin modulates macrophage polarization. (**A**) Representative flow cytometry plots showing the expression of CD86 (M1 marker) and CD206 (M2 marker) on macrophages under different treatment conditions: control, LPS, and LPS + curcumin. (**B**) Quantification of M2 and M1 macrophage populations. Data are shown as mean ± SD; n = 3. ** *p* < 0.01 compared with LPS group; NS not significant.

Flow cytometric analysis demonstrated distinct shifts in macrophage polarization in response to LPS and curcumin treatment. In the control group, macrophages predominantly expressed low levels of CD86 and CD206. Upon LPS stimulation, a marked increase in CD86^+^ macrophages (M1-like) was observed (17.0%), indicating classical pro-inflammatory activation. Co-treatment with curcumin slightly reduced CD86^+^ cells (14.3%) and markedly increased CD206^+^ cells (1.04% vs. 0.20% in LPS alone), suggesting a partial switch toward an anti-inflammatory M2-like phenotype (Figure 6A). Quantitative analysis showed that the percentage of F4/80^+^CD206^+^ cells significantly increased in the LPS + curcumin group compared to both the control and LPS groups (** *p* < 0.01), while the percentage of F4/80^+^CD86^+^ cells remained high following LPS stimulation and was only modestly decreased by curcumin treatment.

### 2.2. Discussion

In this study, we systematically evaluated the protective effects of curcumin, HBC, and their composite Cur@HBC in an ACLT-induced OA model. Histological staining revealed severe cartilage destruction and matrix loss, accompanied by severe synovial inflammation, in the ACLT group. Treatment with either curcumin or HBC alone provided moderate protection; however, the combination of both (Cur@HBC) showed the most pronounced therapeutic effect. The marked improvement in Safranin O and Toluidine Blue staining confirmed that Cur@HBC preserved the proteoglycan-rich cartilage matrix. This was evidenced by the significantly lower Mankin and OARSI scores as well as reduced synovitis scores in the Cur@HBC group, suggesting effective inhibition of OA progress. These findings imply that the composite formulation of Cur@HBC not only enhances curcumin’s bioavailability and targeted delivery, but also synergistically amplifies its anti-inflammatory and cartilage-protective properties.

At the molecular level, the protective effect of Cur@HBC was associated with the maintenance of cartilage homeostasis. Immunohistochemical analysis showed that ACLT-induced cartilage damage was characterized by reduced Collagen II and increased MMP13 expression, reflecting matrix breakdown and catabolic remodeling.

Treatment with Cur@HBC demonstrated superior efficacy, likely due to enhanced bioavailability and targeted delivery. Treatment with curcumin alone exhibited a partial protective effect, consistent with its known anti-inflammatory and antioxidant properties [38]. Interestingly, the HBC carrier without curcumin also exerted protective effects, suggesting that the carrier itself may have cartilage-supportive or lubrication function. These findings support the utility of hydrogel delivery systems for osteoarthritis treatment, particularly in enhancing the efficacy of natural compounds like curcumin.

The imaging results further supported these observations. The significant reductions in bone volume (BV/TV) and joint space narrowing in the ACLT group reflect hallmark pathological changes in OA. Curcumin alone partially attenuated these changes. The Cur@HBC formulation, however, demonstrated superior structural preservation, possibly due to the enhanced local retention and bioavailability afforded by the hydrogel system. Behavioral analysis corroborated the structural findings, showing that Cur@HBC significantly improved joint function, as evidenced by a decreased response time in motor testing. These results suggest that the nanocarrier-based curcumin delivery system not only protects joint integrity, but also restores functional outcomes more effectively than curcumin alone.

To investigate the underlying mechanisms, chondrocyte apoptosis and inflammatory cytokine production were assessed. The substantial reduction in apoptotic cell ratio and IL-6 secretion upon curcumin treatment highlights its potent anti-apoptotic and anti-inflammatory effects. In addition, macrophages are key immune cells that play a central role in the progression and resolution of inflammation in osteoarthritic (OA) joints. Within the synovial microenvironment, macrophages exhibit remarkable plasticity and can polarize into either the classically activated (M1) or alternatively activated (M2) phenotype in response to environmental cues [39]. M1 macrophages, also called classically activated macrophages, mainly exert pro-inflammatory effects, secrete pro-inflammatory cytokines such as IL-1β, TNF-α, and IL-6, and produce reactive oxygen and nitrogen species, thereby contributing to cartilage matrix degradation, synovial inflammation, and pain sensitization. In contrast, M2 macrophages, also referred to as alternatively activated macrophages, exhibit anti-inflammatory effects in the immune response, promoting tissue repair and remodeling through the secretion of IL-10, TGF-β, and arginase-1 [40]. In osteoarthritis, the imbalance between M1 and M2 macrophages favors a chronic inflammatory state, characterized by sustained M1 dominance and reduced M2-mediated resolution. This polarization shift leads to a persistent catabolic environment that accelerates cartilage destruction and subchondral bone remodeling. Numerous studies have demonstrated that modulating macrophage polarization to restore the M1/M2 balance can attenuate joint inflammation; for instance, hyaluronic acid, rapamycin, and quercetin can promote M2 polarization and halt OA progression [39]. Thus, we explored curcumin’s role in modulating macrophage polarization. LPS stimulation induced the classical M1 phenotype characterized by increased CD86 expression, reflecting a pro-inflammatory response. However, co-treatment with curcumin led to a notable increase in CD206 expression, a hallmark of alternatively activated M2 macrophages, and a mild decrease in M1 marker expression [41]. This phenotype shift indicates that curcumin may promote anti-inflammatory macrophage polarization, potentially contributing to tissue repair and inflammation resolution. The significant upregulation of F4/80^+^CD206^+^ cells implies that curcumin not only inhibits LPS-induced inflammation but may also actively reprogram macrophages toward a reparative state. Taken together, these results support the role of curcumin as a regulator of macrophage plasticity, with potential therapeutic value in controlling inflammatory conditions by modulating innate immune responses.

Overall, our findings establish Cur@HBC as a promising therapeutic strategy for preventing joint degeneration and preserving mobility in osteoarthritis models. The formulation effectively preserves cartilage and bone structure, reduces inflammation, modulates immune response, and restores joint function, highlighting its potential as a multifunctional treatment for OA.

## 3. Conclusions

In summary, this study successfully developed an injectable hydrogel (HBC) with thermosensitive properties that is capable of undergoing sol–gel phase transition at physiological temperature. In vitro cytotoxicity assays further confirmed the biosafety of HBC. By incorporating curcumin into this thermosensitive matrix, we constructed a localized drug delivery system that provides sustained drug release and targeted therapeutic action against osteoarthritis. Comprehensive evaluation demonstrated that the Cur@HBC system effectively attenuated OA development. Importantly, curcumin alleviates chondrocyte apoptosis and modulates macrophage polarization, ultimately favoring it as an OA therapy. Taken together, the thermosensitive curcumin-loaded HBC hydrogel offers a promising platform for intra-articular OA therapy, providing a localized, sustained, and minimally invasive treatment.

## 4. Materials and Methods

### 4.1. Preparation and Characterization

Hydroxybutyl chitosan (HBC) was synthesized as previously reported [42] with the following parameters: Chitosan was alkalinized in a 50% (*w*/*w*) NaOH solution for 24 h at room temperature. After the removal of excess alkali, the solid was dispersed in isopropanol/water (1:1, *v*/*v*) for 24 h. 1,2-Butene oxide (200 mL) was then added, and the mixture was stirred at 55 °C for 12 h. The reaction was neutralized to pH 7 via dropwise addition of HCl, and the neutralized mixture was dialyzed extensively against water for 5 days, filtered, and lyophilized to obtain HBC. An aqueous 5 wt% HBC solution was prepared and stored at 4 °C until use. Structural characterization was performed via ^1^H NMR and FTIR spectroscopy.

### 4.2. Drug Release

Curcumin was first dissolved in ethanol and then added to 5 wt% HBC aqueous solution; the dispersion was magnetically stirred at 4 °C for 3 h to yield a uniformly dispersed, injectable thermosensitive formulation with a final curcumin content of 20 µg mL^−1^. For release testing, at predefined times prior to a common endpoint, 200 µL of the drug-loaded gel was placed into 1.0 mL release medium (PBS + 1% Tween-80) in separate tubes under identical conditions; at the endpoint, all tubes were collected simultaneously. The supernatant (100 µL) from each tube was transferred to a 96-well plate and absorbance at 425 nm was recorded on a microplate reader. Curcumin concentration at each time point was calculated from a calibration curve prepared in the same medium, and the results were used to determine the release profile.

### 4.3. Rheological Characterization

Rheology was performed on an Anton Paar rheometer (MCR 302, Anton Paar USA Lnc, Ashladn, VA, USA) in the oscillatory mode to evaluate viscoelasticity and thermoresponsive transition. Hydrogel disks were molded identically for all tests and measured using a parallel-plate geometry (plate diameter = 20 mm; gap = 1 mm). Temperature-sweep experiments were conducted from 4 °C to 40 °C at f = 1 Hz and γ = 1%, and storage (G′) and loss (G″) moduli were recorded as a function of temperature. The phase-transition temperature was determined from the G′/G″ crossover.

### 4.4. Cell Culture

BHK-21 cells were cultured in Dulbecco’s modified Eagle medium (DMEM) (GIBCO, Thermo Scientific, Waltham, MA, USA) containing 10% fetal bovine serum (FBS) (GIBCO, Thermo Scientific, Waltham, MA, USA) and 1% penicillin–streptomycin (P/S, GIBCO, Thermo Scientific, Waltham, MA, USA) at 37 °C with 5% CO_2_. The primary chondrocytes were isolated and cultured from the knee joint cartilage in mice. The cells were cultured in DMEM/F12 (GIBCO, Thermo Scientific, Waltham, MA, USA) containing 20% FBS and 1% P/S at 37 °C with 5% CO_2_. BHK-21 and primary chondrocytes were stimulated with TNF-α (Novoprotein, Suzhou, China) (20 ng/mL) to mimic cellular inflammatory responses in the human body.

### 4.5. Cell Cytotoxicity

The cells were seeded in a 96-well plate at a density of 5 × 10^3^ cells/well and were cultured overnight prior to the addition of a series levels of 10 μg/mL, 5 μg/mL, 2 μg/mL, 1 μg/mL, and 0 μg/mL After 24 h, cytotoxicity assessment was conducted using the Cell Counting Kit 8 assay (CCK8, GLPBIO, Walnut, CA, USA).

### 4.6. Cell Apoptosis

Apoptotic cells were detected using an Annexin V-FITC/propidiumiodide (PI) apoptosis detection kit (KeyGEN BioTECH, Nanjing, China). First, chondrocytes were seeded in a six-well plate at a density of 1 × 10^5^ cells/well and cultured overnight. A total of 5 μg/mL of curcumin was then added to the cell culture medium with or without supplementation of TNF-α at 20 ng/mL. After 24 h, the suspended chondrocytes after enzymatic digestion were washed twice with PBS and then stained with Annexin V-FITC for 10 min at room temperature, followed by PI staining for another 5 min. After that, the cells were analyzed by flow cytometry (Beckman CytoFLEX, Indianapolis, IN, USA). The number of events recorded in the sample was 10,000 cells. Cells with both negative PI and Annexin V were considered to be normal cells, while those with both positive PI and Annexin V were regarded as apoptotic cells. Each test was repeated independently three times.

### 4.7. Cell Proliferation

Chondrocytes were seeded in five 96-well plates at a density of 3.5 × 10^3^ cells per well and incubated overnight at 37 °C with 5% CO_2_. After treating the 96-well plates with 5 μg/mL curcumin in the medium for 0 h, 6 h, 12 h, 24 h, and 48 h, cell proliferation was assessed using a cell counting kit assay (CCK-8, GLPBIO, Walnut, CA, USA).

### 4.8. Inflammatory Factor Tested by ELISA

Second-generation chondrocytes were seeded onto six-well plates at a density of 5 × 10^4^ cells per well. The control group remained untreated; the inflammation-induced group was stimulated with TNF-α (Novoprotein, Suzhou, China) (20 ng/mL) for 24 h after cell attachment; to simulate the inflammatory state of chondrocytes, the treatment group was treated with the drug after inflammation induction and cultured for 24 h. The cell culture supernatant was collected from each group, and an ELISA Kit (MULTI SCIENCES, Hangzhou, China) was used to evaluate the dynamic changes in inflammatory responses after drug treatment.

### 4.9. Macrophage Polarization

Bone marrow cells were isolated from the femurs and tibias of 6-week-old male C57BL/6 mice, followed by culture of the cells in complete DMEM containing 10% fetal bovine serum (FBS, GIBCO, Thermo Scientific, Waltham, MA, USA), 1% penicillin–streptomycin, and 20 ng/mL M-CSF (GIBCO, Thermo Scientific, Waltham, MA, USA) at 37 °C with 5% CO_2_. On day 7, the cells differentiated into mature macrophages. The LPS + curcumin group was pre-treated with 5 μg/mL curcumin for 10 h, followed by treatment with 1 μg/mL LPS for 12 h. Subsequently, TruStain FcX™ (anti-mouse CD16/32) antibody (Biolegend, San Diego, CA, USA) was used to block non-specific binding. Then, anti-mouse CD206 antibody (MULTI SCIENCES, Hangzhou, China) and anti-mouse CD86 antibody (MULTI SCIENCES, Hangzhou, China) were used for staining. The stained individual macrophages were analyzed using a flow cytometer (BD Biosciences, Franklin Lakes, NJ, USA), and the data were analyzed and presented using the FlowJo V.10.8.1 software.

### 4.10. Animal Model

All experimental procedures were carried out in accordance with the guidelines in “the Animal Management Regulations” of the animal research committees of Foshan University Animal Ethics Committee. Thirty-six C57BL/6 (male, 4 weeks old) mice were purchased from the Guangdong Experimental Animal Center (Guangzhou, China). Six animals were randomly assigned to each group. We established a mouse OA model via transection of the anterior cruciate ligaments (ACLT). Two weeks post-surgery, a total volume of 10 μL of saline, HBC, curcumin (20 μg/mL), or Cur@HBC was injected into the knee joint cavity via a microinjector.

For histological staining, knee joint sections were cut at a thickness of 4 μm along the sagittal plane. The degree of destruction of the knee cartilage was evaluated using Safranin O/Fast Green staining (Leagene, Beijing, China), while the Osteoarthritis Research Society International (OARSI) OA histopathology assessment system was applied to assess the severity of cartilage degeneration. Synovitis score was determined by hematoxylin and eosin staining (H&E, Leagene, Beijing, China).

For immunohistochemical staining, the sections were soaked in 3% H_2_O_2_ for 10 min to remove endogenous catalase. The sections were then blocked with serum prior to incubation with the primary antibody overnight at 4 °C. Then, the sections were incubated with the HRP-conjugated secondary antibody for 1 h at room temperature prior to detection by the 3,3 N-Diaminobenzidine Tertrahydrochloride (DAB) visualization method. For immunofluorescence staining, the knee joint sections were first blocked with 5% goat serum and then incubated with primary antibodies overnight at 4 °C. Then, the sections were incubated with fluorescently labeled secondary antibodies for 1 h at room temperature in the dark. The sealed slides were photographed after DAPI staining.

### 4.11. Behavioral Assessment

After the animals were acclimatized to the environment for 30 min, the soles of the left hind limbs were stimulated with a thermal pain detector for a duration of <5 s. If the animals showed rapid licking or lifting of feet, the reaction was recorded as positive; otherwise, it was recorded as negative. The foot retraction time was also recorded. At an interval of 5 min each, the detection ended after data recording for 6 times.

### 4.12. Micro-CT Analysis

Potential changes to the subchondral trabecular bone microarchitecture and mineral density in the region of interest (ROI) beneath the subchondral cortical bone were measured by micro-CT. Briefly, the knee joint of each mouse was isolated after euthanasia, and the surrounding soft tissue was removed. After fixation with 4% paraformaldehyde solution for 24 h, the knee joints were scanned using a micro-CT system (Micro CT NMC-200, NEMO, PINGSENG Hwalther, Kunshan, China), with the scanning parameters set as 90 kV, 90 μA, and 10 μm in spatial resolution.

### 4.13. Statistical Analysis

Data are presented as mean ± standard deviation (mean ± S.D.). Differences between two groups were statistically analyzed using unpaired, two-tailed Student’s *t*-test, while analysis of variance (ANOVA) with Dunnett’s post hoc test was performed to compare data when there were more than two groups of variables. The levels of significance were set at * *p* < 0.05, ** *p* < 0.01, and *** *p* < 0.001. All statistical analyses were performed with GraphPad Prism software, version 8.0 (GraphPad Software, Inc., CA, USA).

## Data Availability

Data will be made available upon request.

## Supplementary Materials

The following supporting information can be downloaded at https://www.mdpi.com/article/10.3390/gels12010007/s1, Video S1: The sol–gel transition of Cur@HBC from 4 °C to 37 °C; Figure S1. SEM micrographs of HBC and Cur@HBC hydrogels. Scale bar: 200 μm; Table S1. Elemental analysis results of chitosan (CS) and hydroxybutyl chitosan (HBC).

## Abbreviations

The following abbreviations are used in this manuscript:

OA	Osteoarthritis
HBC	Hydroxybutyl chitosan
BAPC	Bayesian Age–Period–Cohort
YLD	Years lived with disability
GBD	Global Burden of Disease
NSAIDs	Non-steroidal anti-inflammatory drugs
ROS	Reactive oxygen species
TCM	Traditional Chinese medicine
MOFs	Metal–organic frameworks
SA	Sodium alginate
FBS	Fetal bovine serum
ROI	Region of interest
