# Supplementary material for: A Novel Thermosensitive Curcumin-Loaded Hydrogel That Modulates Macrophage M1/M2 Polarization for Osteoarthritis Therapy
Source: Gels. 2025 Dec 21;12(1):7. doi: 10.3390/gels12010007 (PMC12840833; doi:10.3390/gels12010007)
Supplement: Supplementary file 1 [file gels-12-00007-s001.zip › Supplementary Files/Table S1.docx]

**Elemental Analysis Procedure**

Elemental analysis of carbon, hydrogen, and nitrogen (CHN) was performed to quantify compositional changes before and after hydroxybutyl modification. Samples of chitosan (CS) and hydroxybutyl chitosan (HBC) were dried to constant weight to eliminate residual moisture and then subjected to CHN determination using a standard combustion-based analytical method. The measured C/N ratios were used to estimate the degree of substitution (DS) of hydroxybutyl groups according to established calculations for ether-modified polysaccharides. The DS was calculated according to:

DS =$\frac{{14}/{N_{HBC}}-{14}/{N_{CS}}}{M_{sub}}$

where 14 is the molar mass of nitrogen (per repeat unit), N_CS_ and N_HBC_ are the nitrogen mass fractions of chitosan and HBC, and M_sub_ is the mass added per substitution (for C_4_H_9_O_2_, 89 g/mol). Using the measured values (N%: CS = 7.65%, HBC = 4.76%), the DS was calculated to be approximately 1.25 substituents per repeating unit, consistent with the ^1^H NMR-based estimate.

Table SI-1. Elemental analysis results of chitosan (CS) and hydroxybutyl chitosan (HBC).

|  | N[%] | C[%] | H[%] |
| --- | --- | --- | --- |
| CS | 7.65 | 41.33 | 7.029 |
| HBC | 4.76 | 51.17 | 8.464 |
