# Supplementary material for: A Novel Thermosensitive Curcumin-Loaded Hydrogel That Modulates Macrophage M1/M2 Polarization for Osteoarthritis Therapy
Source: Gels. 2025 Dec 21;12(1):7. doi: 10.3390/gels12010007 (PMC12840833; doi:10.3390/gels12010007)
Supplement: Supplementary file 1 [file gels-12-00007-s001.zip › Supplementary Files/Figure S1.docx]

**SEM Sample Preparation**

HBC and Cur@HBC hydrogels were first allowed to fully gel at 37 ^o^C and subsequently frozen at -80 ^o^C and lyophilized to preserve their internal porous architecture. The resulting dry samples were gently fractured to expose representative cross-sections. Prior to imaging, all specimens were mounted onto conductive stubs and coated with a thin conductive layer to prevent surface charging. SEM micrographs were collected under conditions appropriate for observing the micro-scale morphology and pore structure of the hydrogels.


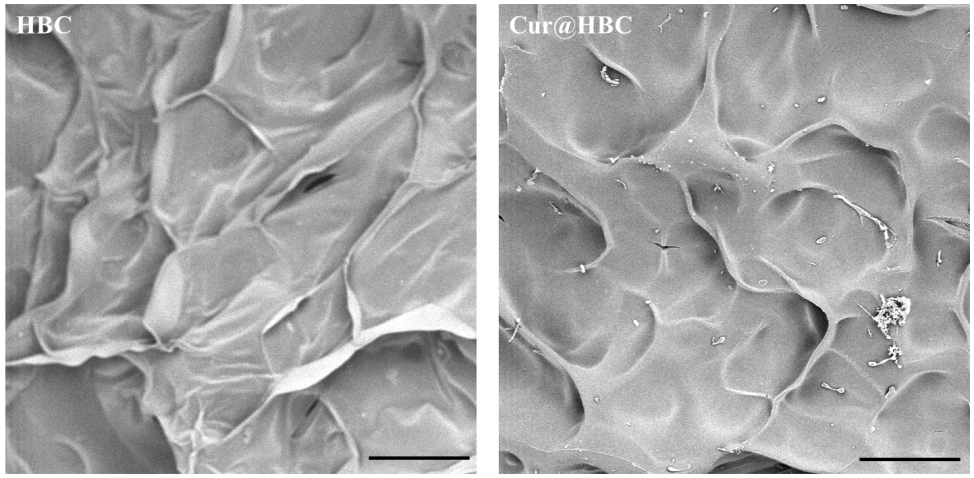


Figure S1. SEM micrographs of HBC and Cur@HBC hydrogels. Scale bar: 200 μm.
